# Breast Implant-Associated Anaplastic Large Cell Lymphoma: A Case Report and Literature Review

**DOI:** 10.7759/cureus.546

**Published:** 2016-03-26

**Authors:** Haley Letter, Baiywo Rop, Michele N Edison, Patricia Turner

**Affiliations:** 1 Diagnostic Radiology, Florida Hospital-Orlando; 2 Radiology, Florida Hospital-Orlando

**Keywords:** anaplastic large cell lymphoma, peri-implant fluid collection, Breast, mri

## Abstract

Introduction

Anaplastic large cell lymphoma is a very rare T-cell lymphoma that has only recently been found to be associated with breast implants. It has been described in the literature mainly in the form of case reports. This article focuses on the imaging characteristics of this rare disease. We hope to increase awareness of breast imagers and referring physicians to improve early detection rates.

Case Report

We present the case of a 32-year-old female who presented with several weeks of pain and firmness in her right breast. MRI and ultrasound demonstrated a peri-implant fluid collection. Ultrasound-guided aspiration revealed anaplastic large cell lymphoma. The patient was treated with implant removal alone and has now been in remission for 3 years.

Conclusion

Anaplastic large cell lymphoma of the breast is a very rare entity that has mainly been described in the literature as case reports. As in the case of our patient, imaging findings can be very non-specific, and it is important for surgeons, breast imagers, and oncologists to be aware of this rare disease to ensure prompt diagnosis.

## Introduction

Anaplastic large cell lymphoma (ALCL) of the breast is a rare entity that has mainly been described in case reports and small case series. In 2011, the FDA issued a warning statement about the association of breast implants with ALCL and at that time had confirmed approximately 60 cases of breast implant-associated lymphoma [1]. Clinical presentation is variable and may include a palpable mass in the breast or axilla, generalized breast pain, or breast firmness. Given the rarity of this disease and lack of awareness by physicians, diagnosis is often delayed. Imaging plays a valuable part in the work up of these patients, but the imaging characteristics are rather non-specific. As the number of patients undergoing breast augmentation or reconstruction increases, heightened awareness of ALCL by primary physicians, surgeons, and breast imagers is critical to ensure prompt diagnosis and treatment. 

## Case presentation

**Figure 1:**
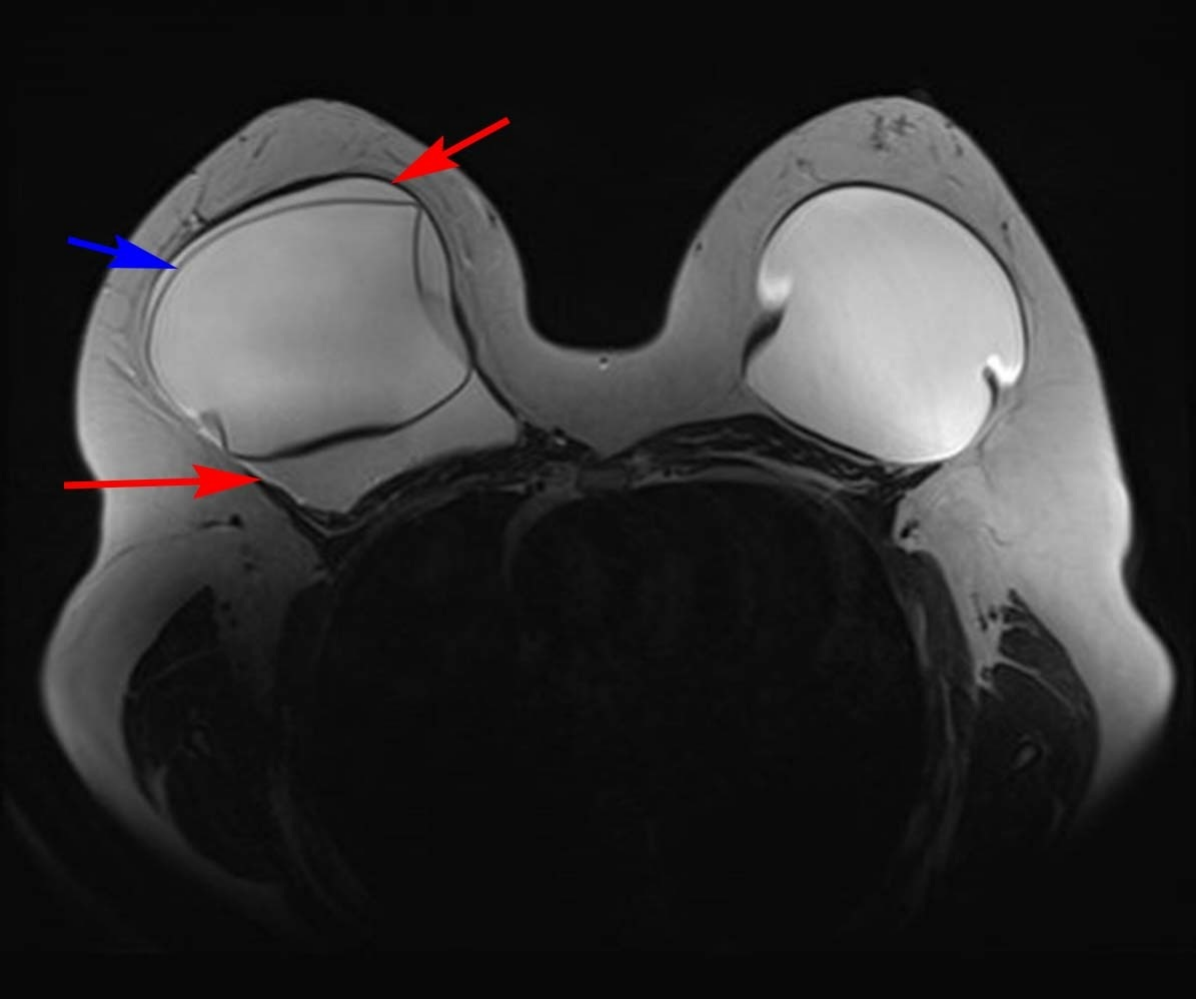
Axial MRI T2 weighted image of the bilateral breasts A T2 hyperintense collection (red arrows) surrounds the implant in the right breast. The implant capsule (blue arrow) is intact.

**Figure 2:**
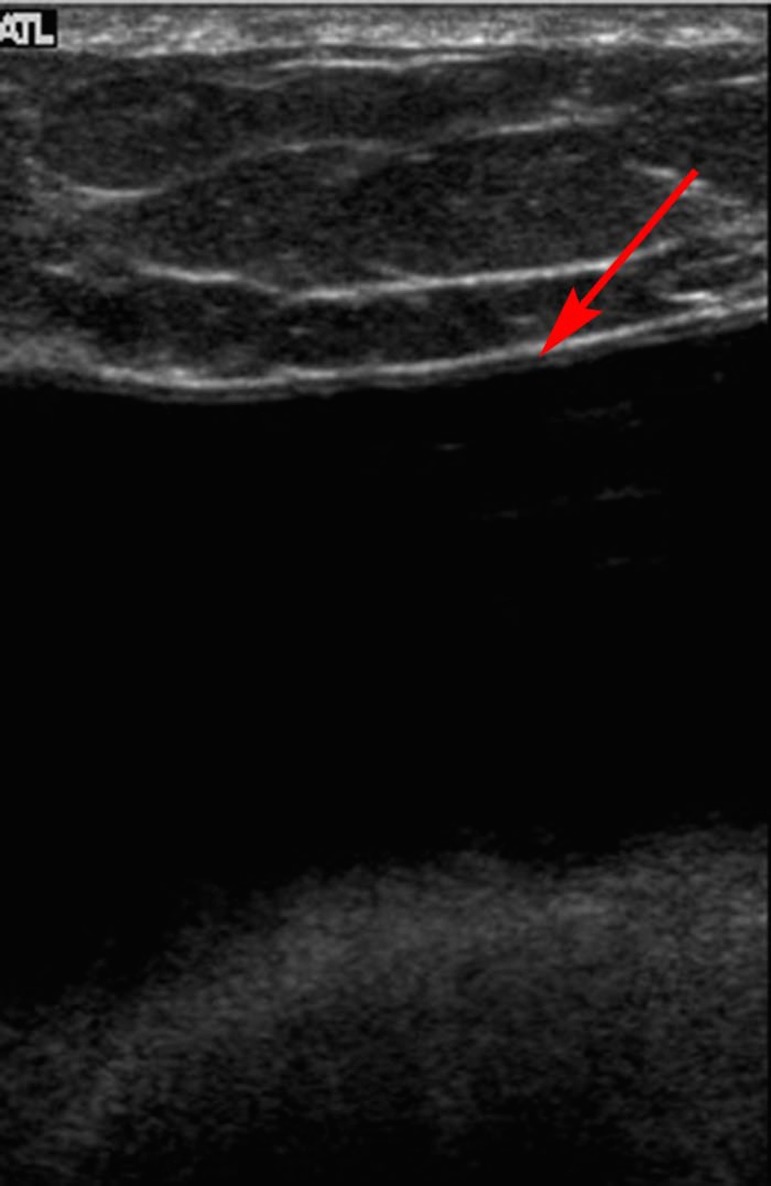
Gray scale ultrasound image of the right breast A simple fluid collection (red arrow) is seen superficial to the underlying implant.

A 32-year-old Hispanic female presented with pain and firmness in her right breast for several weeks. She had undergone breast augmentation with saline implants 16 years prior to presentation. Routine laboratory work revealed no abnormalities. She was referred by her primary care physician for MRI of the right breast. MRI demonstrated a large fluid collection surrounding the right breast implant (Figure 1) with no associated significant enhancement. There was no suspicious mass lesion and no axillary adenopathy. The implant was intact. An ultrasound was performed and demonstrated a simple fluid collection surrounding the implant (Figure 2). Again, no mass was identified. Ultrasound-guided fluid aspiration was performed which yielded approximately 20 cubic centimeters of yellow, serous fluid. Pathology demonstrated a seroma-associated anaplastic large cell lymphoma of the right breast. Anaplastic lymphoma kinase (ALK) gene testing was negative.

The patient underwent PET/CT which revealed no metastatic disease. She was taken to surgery, and both implants were removed. She did not receive neoadjuvant or adjuvant chemotherapy or radiation therapy. The patient has now been disease-free with no evidence of disease recurrence for three years. 

## Discussion

Anaplastic large cell lymphoma (ALCL) of the breast is a very rare T-cell lymphoma that has been found to be associated with breast implants. Lymphoma of the breast in general is very rare and represents only 0.4% to 0.5% of breast cancers and 1% to 2% of lymphomas [2]. There is no difference in disease incidence between patients with saline versus silicone implants [3]. There is also no difference identified in the number of patients with ALCL among women who received implants for cosmetic reasons versus breast reconstruction following mastectomy [3].

The clinical presentation of implant-associated ALCL can be variable, but most often presents as pain and/or rapid swelling of the breast [4]. An appropriate history and physical exam are imperative to exclude other (much more common) causes of breast swelling and pain, such as trauma or infection [3]. Nonetheless, a patient with these symptoms should be referred for imaging of the breast.

A diagnostic mammogram is usually the first imaging study performed to evaluate a patient with vague complaints of breast swelling or pain. Ultrasound is performed subsequently and interpreted in conjunction with the mammogram. The imaging findings of ALCL are often very non-specific. Approximately two-thirds of patients present as our patient did, with a peri-implant fluid collection detected on imaging [3]. The differential diagnosis for peri-implant fluid collection includes infection, inflammation, idiopathic, implant rupture, seroma, hematoma, malignancy, and gel bleed (in the setting of silicone implants). One-third of patients with ALCL will have a defined mass lesion in the breast [3]. Ultrasound is a very cost effective test and has the advantage of being used for image-guided aspiration [5]. MRI is also a very sensitive test for evaluating findings associated with suspected lymphoma, including effusion or mass. It is also very sensitive for evaluation of implant integrity [3]. One retrospective review of imaging in ALCL of the breast demonstrated a similar sensitivity between ultrasound and MRI in detecting peri-implant fluid, with sensitivities of 84% and 82%, respectively [5]. The sensitivity of mass detection by ultrasound and MRI was 46% and 50%, respectively [5]. Mammography demonstrated a sensitivity of only 73% in detecting an abnormality and was not able to distinguish between a fluid collection or a mass. [5]. Peri-implant fluid can be a normal finding in many cases; however, in the setting of an enlarging collection that occurs late in the peri-operative period, lymphoma should be considered in the differential. A consensus statement by a group of plastic surgeons recommends ultrasound-guided fluid aspiration of any enlarging peri-implant fluid collection that occurs greater than one year following implant placement [6]. At our institution, we routinely send all aspirated peri-implant fluid for cytology, gram stain, culture, and sensitivity. 

Breast implant associated ALCL has a unique histopathological identity. Most primary breast lymphomas are of B-cell lineage, while all reported cases of breast implant-associated ALCL have been of T-cell lineage [3]. There are two unique types of implant-associated ALCL, “in situ” and “invasive” [7]. A retrospective review of ALCL cases associated with implants demonstrated that the in situ type is much more indolent and has a very good overall survival with implant removal only. The invasive type can be more aggressive and may require systemic therapy in addition to implant removal. One retrospective study demonstrated that, at a minimum, complete capsulectomy and implant removal is required for all cases of implant-associated ALCL and adjuvant therapy is considered on a case-by-case basis [8]. 

## Conclusions

Breast implant-associated ALCL is a very uncommon, newly-described disease that has been documented mainly in case reports. Much is still unknown about the pathogenesis and epidemiology of the disease. Given the rarity, the imaging features of this disease may be less readily recognized, and this can lead to a delay in diagnosis. It is imperative for breast imagers to consider this entity in patients with breast implants who present with a delayed-onset enlarging peri-implant fluid collection. Ultrasound and MRI are the two most sensitive tests for detection of peri-implant fluid collections, and ultrasound has the added benefit of being readily available for image-guided aspiration and diagnosis. 
